# Transcriptional profile of oil palm pathogen, *Ganoderma boninense*, reveals activation of lignin degradation machinery and possible evasion of host immune response

**DOI:** 10.1186/s12864-021-07644-9

**Published:** 2021-05-05

**Authors:** Braham Dhillon, Richard C. Hamelin, Jeffrey A. Rollins

**Affiliations:** 1 Department of Plant Pathology, University of Florida, Fort Lauderdale Research and Education Center, Davie, FL 33314 USA; 2 Department of Forest and Conservation Sciences, Faculty of Forestry, University of British Columbia, Vancouver, BC Canada; 3 Department of Plant Pathology, University of Florida, 1453 Fifield Hall, Gainesville, FL 32611-0680 USA

**Keywords:** Ganoderma, Boninense, Plant-pathogen interaction, Differentially expressed genes, White-rot, Oil palm, RNA-seq, Transcriptome

## Abstract

**Background:**

The white-rot fungi in the genus *Ganoderma* interact with both living and dead angiosperm tree hosts. Two *Ganoderma* species, a North American taxon, *G. zonatum* and an Asian taxon, *G. boninense*, have primarily been found associated with live palm hosts. During the host plant colonization process, a massive transcriptional reorganization helps the fungus evade the host immune response and utilize plant cell wall polysaccharides.

**Results:**

A publicly available transcriptome of *G. boninense* - oil palm interaction was surveyed to profile transcripts that were differentially expressed *in planta*. Ten percent of the *G. boninense* transcript loci had altered expression as it colonized oil palm plants one-month post inoculation. Carbohydrate active enzymes (CAZymes), particularly those with a role in lignin degradation, and auxiliary enzymes that facilitate lignin modification, like cytochrome P450s and haloacid dehalogenases, were up-regulated *in planta*. Several lineage specific proteins and secreted proteins that lack known functional domains were also up-regulated *in planta*, but their role in the interaction could not be established. A slowdown in *G. boninense* respiration during the interaction can be inferred from the down-regulation of proteins involved in electron transport chain and mitochondrial biogenesis. Additionally, pathogenicity related genes and chitin degradation machinery were down-regulated during the interaction indicating *G. boninense* may be evading detection by the host immune system.

**Conclusions:**

This analysis offers an overview of the dynamic processes at play in *G. boninense* - oil palm interaction and provides a framework to investigate biology of *Ganoderma* fungi across plantations and landscape.

**Supplementary Information:**

The online version contains supplementary material available at 10.1186/s12864-021-07644-9.

## Background

The *Ganoderma* genus in the order Polyporales contains laccate (shiny) shelf fungi found in temperate and tropical forests and urban landscapes. *Ganoderma* is a diverse genus of wood decay fungi, with both opportunist species that grow on decaying or dead wood, and pathogenic species that attack and kill trees. Wood-decay fungi exist across a degradation continuum depending on the plant cell wall components they decompose [1]. Brown-rot fungi breakdown cellulose but cannot metabolize lignin, whereas, white-rot fungi, like, *Ganoderma*, carry the necessary enzymes needed to mineralize lignin, in addition to enzymes that degrade cellulose and hemicellulose [2].

As with other macrofungi, species of *Ganoderma* were traditionally described based on their macromorphology, host and geography [3–5]. In North America, 13 species of *Ganoderma* were resolved molecularly [6], with additional species recognized in Europe and Asia [7]. Multilocus phylogeny clustered *Ganoderma* taxa into three clades that were not constrained by geographical origin [6, 7]. Clade C contains two species, *G. zonatum*, native to North America and its sister species from Asia, *G. boninense*. Both species are found in sub-tropical and tropical regions and have been collected from the monocot plant host, palms, family Arecaceae.

Palms are ‘the’ iconic plant species of the tropics. There are ~ 2600 species of palms that dot the landscape in tropical and sub-tropical ecosystems. Palms are an important part of the social and economic spheres, both globally and locally. Even though palms are well-known for three edible products i.e. coconuts, dates and oil, they are a source for other commodities like palm syrup, nuts, jams/jellies, wine, dyes, carnauba wax, rattan cane and wood. In 2018, palm oil accounted for 40.2% of the world’s vegetable oil production, with 85% of the total palm oil being produced in Malaysia and Indonesia [8]. In 2016, an industry based on one commodity, palm oil, generated 2.9 million jobs world-wide and contributed $39 billion to the global GDP [9].

*G. boninense*, a soil-borne fungus that colonizes its host through the roots [10], poses a serious threat to oil palm plantations in southeast Asia [11]. Similarly, *G. zonatum* has been described as a pathogen on palms in the southeastern US [12]. Both *Ganoderma* species associate with palms and may share mechanisms that facilitate palm tissue colonization. Genomic and transcriptomic analyses and comparisons can help us understand these mechanisms and develop better control methods. To understand the gene expression changes that occur in the white-rot fungus *G. boninense* as it interacts with its oil palm host, *Elaeis guineensis,* a publicly available RNAseq dataset was obtained from GenBank and analyzed. The objective was to identify the genes that are differentially expressed during the interaction and allow *G. boninense* to colonize and cause disease in its palm host. Insights into the mechanics of *Ganoderma*-palm interaction gained from *G. boninense* pathosystem will inform and address questions related to the biology and disease ecology of *G. zonatum*.

## Results

**Fig. 1:**
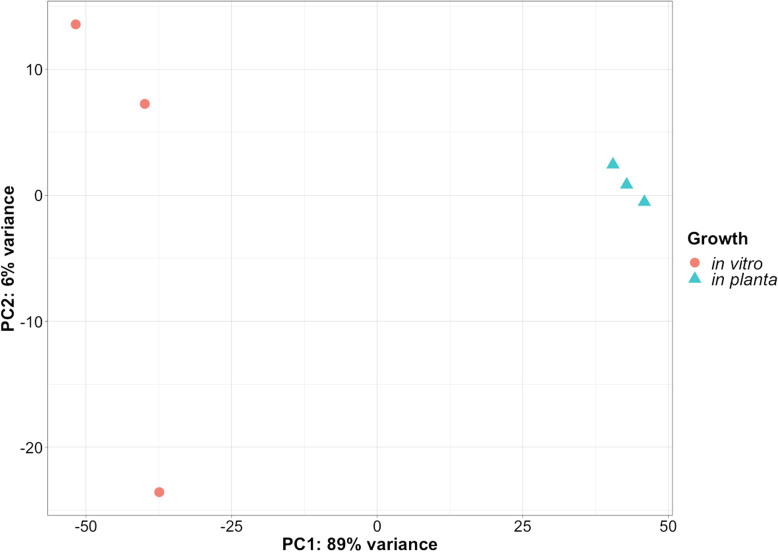
Principal component analysis of six *Ganoderma boninense* samples used for RNAseq. Principal component analysis (PCA) was conducted using transformed normalized read counts for 15,536 transcript loci. The three replicates for each group, fungal growth in vitro and *in planta*, are color coded red and green, respectively

The *Ganoderma boninense* genome [13] and *G. boninense* - *Elaeis guineensis* interaction transcriptome [14], were obtained from NCBI and analyzed. An average of 156 and 131 million RNAseq reads were generated from three biological replicates for the two growth conditions: (1) in vitro, i.e., *G. boninense* grown on artificial media, and (2) *in planta*, i.e., *G. boninense* interacting with roots of oil palm seedlings, respectively. The reads from the in vitro (82%) and *in planta* (57%) growth conditions that mapped to the *G. boninense* genome (Table 1) were assembled to generate a dataset of 15,536 loci. A total of 1560 differentially expressed genes (DEGs; absolute value [log_2_FC > 2]; p_adj_ < 0.05) were identified with 669 and 891 transcripts that were up- and down-regulated, respectively, *in planta* when compared to the artificial media (Additional file 1: Table S1). Principal component analysis using the transformed normalized counts showed that the first component explained 89% of the variation and clearly separates the transcriptome samples derived from in vitro and *in planta* growth conditions (Fig. 1). The three replicates within the *in planta* group had low variation, with some separation of the *G. boninense* transcriptome obtained from samples grown in vitro along the second principal component (Fig. 1).

**Table 1:** Mapping statistics of the reads aligned to the *Ganoderma boninense* genome

	Reads (Total)	Reads (Mapped)	Percent mapped
In vitro (Rep1)	136,217,626	113,700,928	83.5
In vitro (Rep2)	167,719,900	141,320,277	84.3
In vitro (Rep3)	165,359,157	133,937,580	81.0
**In vitro (Avg)**	**156,432,228**	**129,652,928**	**82.9**
*In planta* (Rep1)	163,887,796	88,314,747	53.9
*In planta* (Rep2)	83,977,832	44,634,655	53.2
*In planta* (Rep3)	146,185,776	95,274,605	65.2
***In planta*** **(Avg)**	**131,350,468**	**76,074,669**	**57.4**

**Fig. 2:**
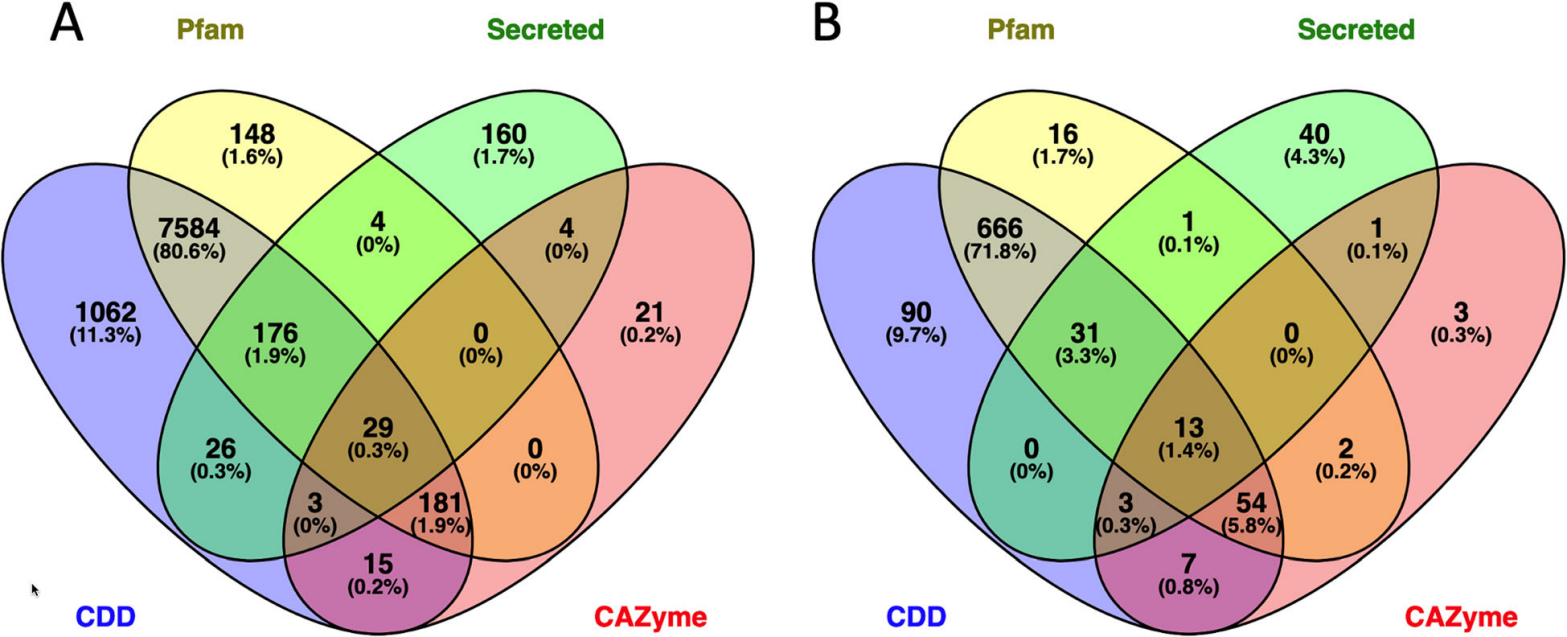
Number of transcripts and differentially expressed genes that were annotated. Venn diagram shows the overlap between database sources for annotated **a** transcript loci and **b** differentially expressed genes. CDD - Conserved Domain Database (NCBI), Pfam - Protein family database, Secreted - proteins with secretion signal, CAZyme - Carbohydrate Active enZymes

The assembled loci and derived proteins were annotated by comparing the sequences with different databases (Fig. 2). A search against the Pfam and NCBI Conserved Domain Database (CDD) databases using the Reverse Position-Specific BLAST (RPS-BLAST) [15], also known as CD-search (Conserved Domain Search), identified protein domains in 59.4% (9228) of the assembled transcript loci (Fig. 2a). Although Pfam models contribute to the NCBI-CDD, the use of Pfam database provided annotation for an additional ~ 1.5% of the total as well as differentially expressed transcripts (Fig. 2a, b). Secreted proteins (402, 4.5%) and CAZymes (253, 2.8%) were identified from predicted protein coding regions (8899) extracted from the RNAseq transcript loci with domain annotations being available for 58.8% of the secreted proteins and 90.1% CAZymes (Fig. 2a). Approximately 72% (1119) of the transcript loci that comprise the *in planta* DEGs dataset had an annotation and 55.2% of the secreted DEGs and 90.8% of the CAZyme DEGs contained annotated protein domains (Fig. 2b).

### CAZymes and other genes involved in lignocellulosic degradation

**Fig. 3:**
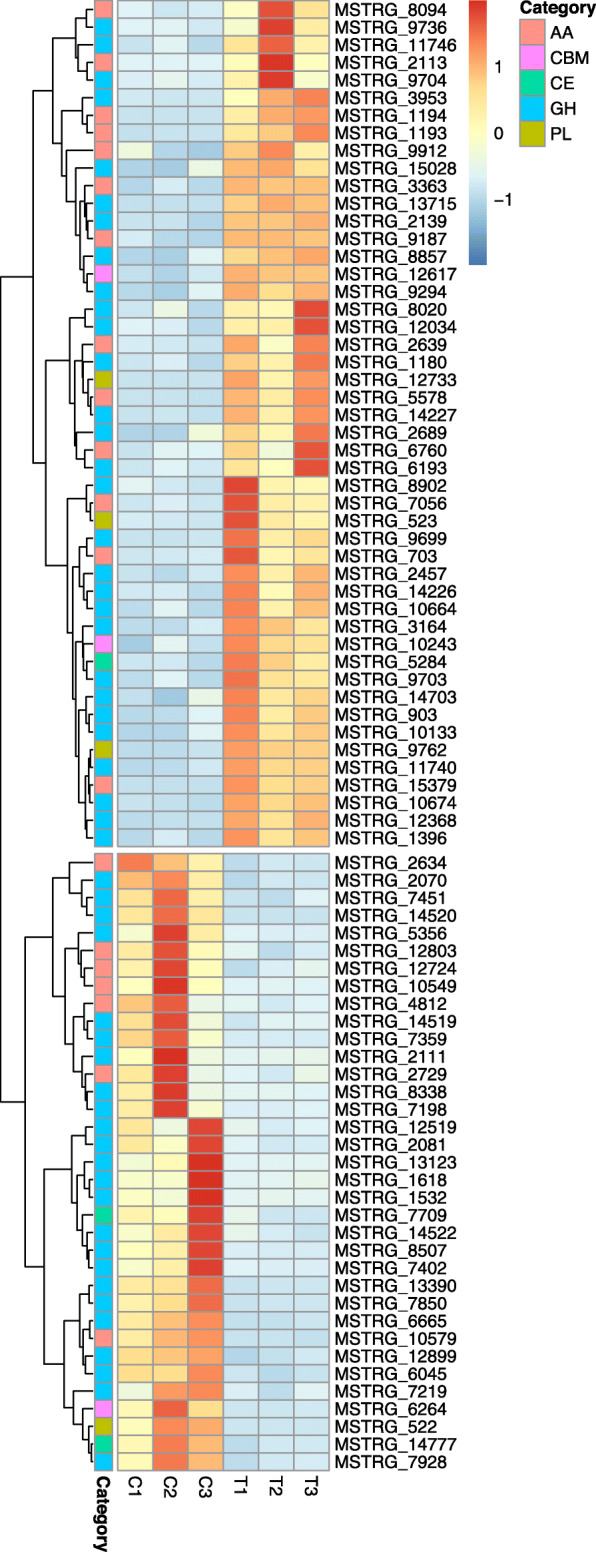
Heatmap for CAZymes that were differentially expressed *in planta*. The expression pattern of CAZymes belonging to five families, auxiliary activities (AA), carbohydrate binding modules (CBM), carbohydrate esterases (CE), glycoside hydrolases (GH) and polysaccharide lyases (PL) is shown here. Columns labelled as C and T represent the two growth conditions, i.e., *G. boninense* growing in vitro on artificial medium, and *G. boninense* interacting with oil palm host (*in planta*), respectively. Gene expression level, expressed as Z-scores calculated from normalized expression values, for each gene is color coded, with red and blue being up- and down-regulated genes, respectively. Rows (genes) were clustered hierarchically

Various classes of carbohydrate active enzymes (CAZymes) were differentially regulated during the *G. boninense* - oil palm interaction compared to in vitro growth on artificial medium (Fig. 3). The auxiliary activities (AA) family of CAZymes that are present in white-rot fungi showed increased expression as the fungus *G. boninense* was colonizing the root tissue of its oil palm host. CAZymes belonging to six families of AA enzymes that specialize in utilizing lignocellulosic substrates and associated byproducts were up-regulated: five multicopper oxidases (AA1_1), four glucose-methanol-choline (GMC) oxidoreductases (AA3), one each of peroxidase (AA2), copper radical oxidase (AA5), and benzoquinone reductase (AA6). Additionally, an enzyme with copper-dependent lytic polysaccharide monooxygenase (LPMO) activity that belongs to the AA9 (formerly GH61) family also showed higher expression in *G. boninense* during its interaction with the plant host.

CAZymes may contain additional non-catalytic modules, carbohydrate binding modules (CBMs), that aid in binding to carbohydrates. At least two enzymes that contain the fungal specific cellulose binding CBM1 domain and one enzyme with galactose binding CBM51 domain were up-regulated as *G. boninense* was utilizing carbon from its oil palm host.

Another family of CAZymes called glycoside hydrolases (GH) target the cellulosic, hemicellulosic, and pectic compounds in plant cell walls. In the interaction between *G. boninense* and its host, enzymes involved in hemicellulose breakdown were up-regulated, including two xyloglucan hydrolases (GH16 or endo-1,3(4)-β-glucanase EC 3.2.1.6) that target cereal D-glucans, one α-glucosidase (GH31), two β-galactosidases (GH35, GH35-CBM51), one GH1, six GH3 including two GH3s that are secreted and two α-glucuronidases (GH115, secreted GH115-CE15) that remove glucuronic acid residues from xylans were up-regulated. CAZymes in the GH family that target pectins (including two GH28, one GH43, one GH51, one CE12), and two β-glucuronidases (GH79) were also up-regulated. Additionally, four α-mannosidases (one each belonging to GH families GH38, GH47, GH92, and GH125) showed elevated expression in *G. boninense* during its interaction with oil palm root tissue.

Two pectate lyases (PL3), one secreted PL8 protein and one alginate lyase domain containing protein from the CAZyme polysaccharide lyase (PL) family of enzymes were also up-regulated during the interaction.

### Oxalic acid biosynthesis and degradation

In the *G. boninense* transcriptome, two copies of oxalate decarboxylases (ODCs) were significantly up-regulated *in planta*. In fact, these two ODCs were among the top 20 most up-regulated DEGs. A third copy of ODC was up-regulated while *G. boninense* was growing on the artificial medium. Two putative copies of oxaloacetate acetylhydrolase, a gene responsible for oxalate biosynthesis, were identified in the *G. boninense* genome but were not differentially expressed during the interaction. Four copies of formate dehydrogenases (FDHs) that degrade formate were differentially expressed. One FDH copy was up-regulated as *G. boninense* was interacting with the oil palm host, whereas three copies were up-regulated during growth on the artificial medium.

### Microbe-associated molecular patterns (MAMPs)

Chitin, a major component of fungal cell walls, also acts as a potent microbe-associated molecular pattern (MAMP) that can be recognized by the plant immune system. A chitin synthase (CHS), chitinase and endochitinase were up-regulated during the *G. boninense* - oil palm interaction. Fungal genes involved in chitin breakdown, including five chitinases, two beta-glucanases and one beta-acetyl hexosaminidase [16], were down-regulated during the interaction. Another modular chitinase that contained a PX domain was also up-regulated during the interaction. A PX domain carrying chitinase protein described earlier in *Phanerochaete chrysosporium* was shown to be expressed but chitinolytically inactive [17]. Furthermore, four *G. boninense* proteins with a CBM50 domain, also known as chitin binding LysM domain, were down-regulated during this interaction.

Additionally, the expression of pathogenicity related genes known from other pathosystems was analyzed in this interaction. A secreted protein belonging to a class of lectins called the ricin B-like (R-type, β-trefoil) lectin, another putative MAMP, was down-regulated in this interaction. Thirteen genes belonging to four gene families, including four hydrophobins, three cerato-platanins, five thaumatin-like proteins and three ferric reductases were down-regulated. Similarly, three GPI-anchored domain proteins, that are localized to the cell wall and involved in signaling pathways in cell wall biogenesis and virulence [18–20], were down-regulated in *G. boninense*-oil palm interaction.

In *G. boninense*, 25 cytochrome P450 transcripts were up-regulated during interaction with the host. Two copies of carboxylesterase enzymes, that have been classified as pathogenicity related genes in *A. mellea* [21], were also up-regulated in *G. boninense in planta*. Moreover, two copies of Hce2 homologs from *G. boninense* were determined to be up-regulated *in planta* (log_2_FC = 7.76, p_adj_ = 2.61E-35; log_2_FC = 6.03, p_adj_ = 1.03E-19).

### Secreted and lineage-specific proteins

**Fig. 4:**
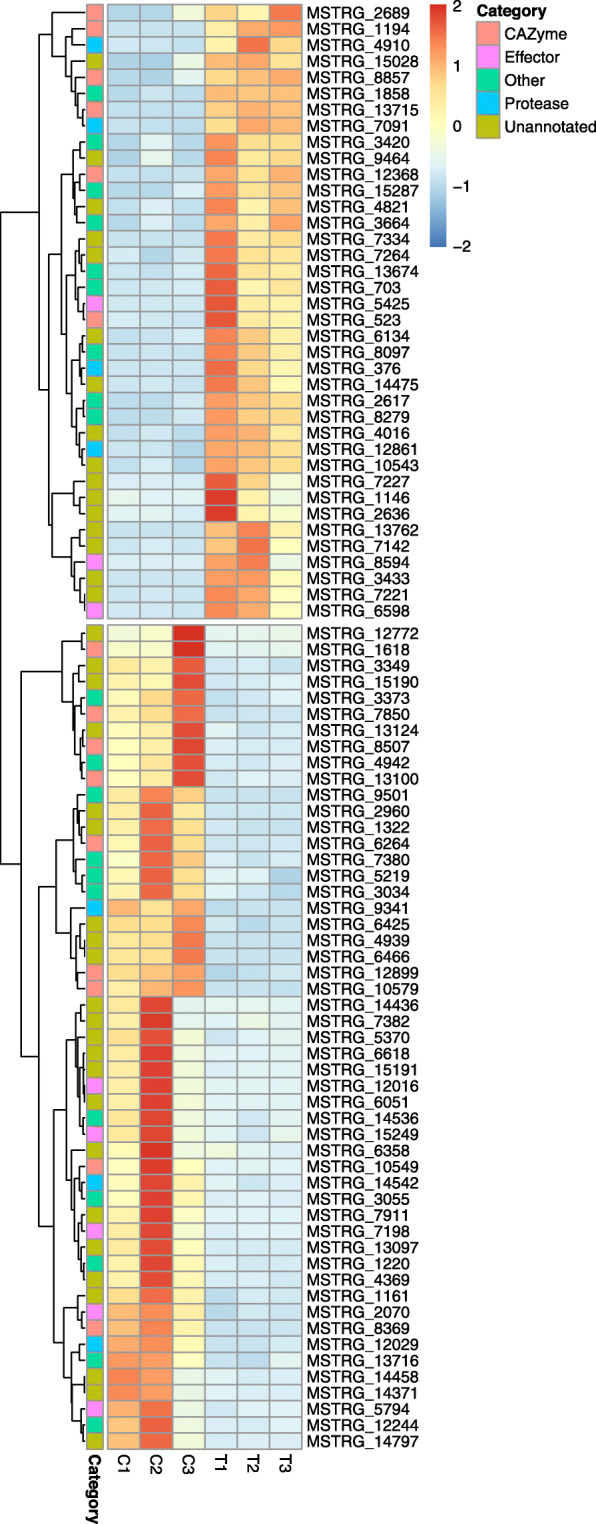
Heatmap for secreted proteins that were differentially expressed *in planta*. Differentially expressed secreted proteins were grouped in to five categories, CAZymes, effectors, proteases, others and unannotated. Columns labelled as C and T represent the two growth conditions, i.e., *G. boninense* growing in vitro on artificial medium, and *G. boninense* interacting with oil palm host (*in planta*), respectively. Gene expression level, expressed as Z-scores calculated from normalized expression values, for each gene is color coded, with red and blue being up- and down-regulated genes, respectively. Rows (genes) were clustered hierarchically

A total of 89 (22.1%) of the predicted secreted proteins have an altered expression pattern when *G. boninense* interacts with palm tissue (Fig. 4), including 38 proteins up-regulated *in planta*. Six secreted proteins were identified as lineage-specific based on lack of similarity to proteins in the NCBI nr database and no known Pfam domains. Three of the secreted lineage-specific proteins were in the top-20 most up-regulated genes during the interaction. One of the most highly up-regulated genes (log_2_FC = 12.03, p_adj_ = 1.37E-71) is a secreted protein that carries the CFEM domain, which is unique to fungi [22]. Among the up-regulated secreted proteins four were identified as hypothetical proteins and eight proteins had domains characteristic of CAZymes.

### Peptidases, transporters and other genes

Peptidases are involved in various activities ranging from housekeeping to plant-pathogen interaction. Four S10 (carboxypeptidase Y) including one secreted S10, and three other serine peptidases, S8 (subtilisin), S24 (LexA repressor), and S53 (sedolisin), were up-regulated *in planta*. Eleven aspartyl peptidases (APs) including two secreted APs, and five metalloproteases including two M48 (STE24), one each of M1 (membrane alanyl aminopeptidase), M20 (glutamate carboxypeptidase), and M24 (methionyl aminopeptidase 1), were up-regulated in *G. boninense* during the interaction. Two protease families, S8 and M1, are two of the most common peptidase families found in fungi [23]. Additionally, one amino acid permease and two oligopeptide transporters were up-regulated during the interaction.

Major facilitator superfamily (MFS1) has been associated with pathogenicity and other metabolic roles in fungi [21]. Seventeen MFS transporters with different Pfam domains, including six MFS1, four MFS_FEN2_like copies, one MFS_FucP_MFSD4_like, and one MFS_MFSD8 were up-regulated during interaction in *G. boninense*, whereas, only three ABC transporters were found to be up-regulated *in planta*.

Among other hydrolases, four copies of lipid metabolizing enzymes or lipases and enzymes involved in benzene-ring metabolism, i.e. haloacid dehalogenases (HAD; five copies) were up-regulated *in planta*. Both these classes of enzymes were up-regulated during woody tissue colonization in the white-rot fungus, *P. chrysosporium* [24].

### Cellular respiration and carbon catabolite repression

All 33 copies of the enzymes that are key in oxidative phosphorylation steps of respiration, including cytochrome b, cytochrome c, and cytochrome-related assembly proteins were down-regulated in *G. boninense* during infection. Transcripts for other enzymes, such as ATP synthases (seven copies) and those carrying the CoA binding domain were down-regulated as well. The alternative oxidase (AOX), a part of the electron transport chain was also down-regulated during the interaction. The proton antiporters (six copies) that are the proton conducting membrane transporters were also down-regulated. Moreover, transcripts of genes in the mitochondrial protein import system that are involved in mitochondrial biogenesis, mitochondrial carrier proteins, and mitochondrial translation activator, MAM33, were all down-regulated during the interaction as well.

The lack of abundant glucose would derepress cell wall degrading enzymes in a process known as carbon catabolite repression [25, 26]. Consistent with this regulation, a majority (80%) of the *G. boninense* glycosyl hydrolases that target plant cell wall components were down-regulated during growth on artificial medium.

## Discussion

Our analyses of *G. boninense* transcriptome as it colonizes its oil palm host revealed that genes in multiple biochemical pathways were differentially expressed early during the interaction, primarily those involved in breakdown and assimilation of metabolites and complex polysaccharides of plant origin, fungal biomass accumulation, generation of microbe-associated molecular patterns (MAMPs) and extracellular lineage-specific proteins, and cellular respiration.

Several transcripts with a role in pathogenesis such as, cerato-platanins, thaumatin-like proteins, ricin B-like lectin, hydrophobins, and ferric reductases were down-regulated in this interaction. Contrary to what was observed in *G. boninense* - oil palm interaction, proteins from three gene families, hydrophobins, cerato-platanins and fungal pathogenesis-related CAP protein, were up-regulated during the basidiomycete pathogen *Moniliophthora roreri* interaction with its host, cacao [27]. Host plants can sense cerato-platanin that is either extracellular or present in the fungal cell wall and mount a defense response [28]. Down-regulation of these MAMPs, that have the potential to trigger a host immune response, lends support to the hypothesis that while maintaining stealth growth, *G. boninense* can stay undetected and evade recognition by the host.

Besides their role in pathogenicity, these proteins play roles in diverse molecular processes. In fact, three classes of secreted proteins, i.e., cerato-platanin, thaumatin and ricin B-like lectin, were reported to be developmentally regulated in basidiomycetes during fruiting body formation [29]. Proteins with cerato-platanin domain were involved in host-pathogen interactions, but these are not necessarily virulence factors [28]. The carbohydrate binding ricin B-like lectins isolated from mushrooms have been studied as nematotoxins or entomotoxins [30] but their role in plant pathogen interaction has not yet been elucidated.

Similarly, other pathogenicity related proteins, such as, CFEM, Hce2, cytochrome P450s and carboxylesterases, that were up-regulated at the sampled infection time-point also play a role in other diverse processes in fungi. Homologs of *C. fulvum* Ecp2 (Hce2) are putative effectors found in multiple fungal species that have possible roles in stress response and adaptation to new ecological niches [31]. Besides its role in lignocellulose degradation, the cytochrome P450 gene family has also been catalogued with pathogenicity related function in *Armillaria mellea* [21]. CFEM domain proteins are involved in a number of different activities, from host-pathogen interaction in *Magnaporthe oryzae* [32, 33] and *Candida albicans* [34] to cell wall biogenesis in *Saccharomyces cerevisiae* [35, 36].

During normal growth, chitin biosynthesis genes like chitin synthases (CHS) and endochitinases [37] are needed for hyphal development and maintenance of cell wall integrity in fungi [38]. The observed up-regulation of CHS and endochitinases suggests that the fungus is growing and increasing in biomass during the interaction. On the other hand, products of chitin breakdown, chitin oligosaccharides, can be recognized by the plant LysM motif containing receptor proteins and elicit immune response [39]. Fungal LysM proteins sequester chitin oligosaccharides thereby preventing detection by the host immune system, as demonstrated for LysM effector Ecp6 from the tomato pathogen, *Fulvia fulva* [40]. The LysM domains in fungi may also interact with chitin from other fungal mycoparasites [41]. Besides chitin, LysM domains may also bind another related molecule, peptidoglycan, that is extensively present in bacterial cell walls [42]. Therefore, the four down-regulated LysM proteins might function in ecological niches where *G. boninense* has to contend with other fungal or bacterial competitors, like those encountered in the soil.

The RNAseq data acquired in this single time-point pilot study provides a snapshot of the differentially expressed transcripts during *G. boninense*-oil palm interaction. In artificial inoculation studies, *Ganoderma* colonized rubberwood blocks are introduced in the palm rhizosphere to initiate root infection. The length of time it takes for *G. boninense* to colonize and kill the host depends on a number of factors, including, the amount of inoculum, proximity of inoculum to the roots, the soil temperature and plant age [10]. Even though younger palms can survive for 6–24 months after infection, foliar symptoms are visible at 2 months post inoculation [10]. This suggests that the process of host colonization initiated by *G. boninense* would be fairly advanced at 4 weeks after inoculation, the time point when samples were collected in the study analyzed here.

A wide range of conditions have been used to understand the transcriptomic response of *Ganoderma* species to different treatments but only one dataset [14] i.e. the one analyzed in this study, had replicated measures of the transcriptional changes that occur during *G. boninense*-palm interaction. Early time points of 3-, 7- and 11-days post inoculation were used to understand the host response in *G. boninense*-oil palm interaction (NCBI BioProject PRJEB27915; [43]). However, wood decay fungi act slowly, and it is likely that early sampling of the interaction might not reveal much about the transcriptional changes occurring during host colonization. RNAseq datasets that comprise of a single time-point at 4 weeks are more likely to reveal components of the host colonization process than multiple early time-points. For white-rot fungi like *Moniliophthora perniciosa*, responsible for witches’ broom on cacao, a longer time frame of 30 days was used for RNAseq to document the transcriptional responses during host colonization [44]. Two additional datasets, one from roots (NCBI BioProject PRJEB7252 [45];) and the other from leaves (NCBI BioProject PRJEB17971 [46];) also document the *G. boninense*-palm interaction, but all replicates per treatment were pooled before sequencing. Thus, the dataset analyzed here appears optimal for capturing the genes and processes important for *G. boninense* colonization of its palm host.

The presence of simple sugars can suppress the expression of fungal genes involved in breakdown and uptake of complex sugars in a process known as carbon catabolite repression (CCR). Addition of glucose decreased the secretion of lignocellulolytic enzymes in *Pleurotus ostreatus* [25]. The existence of CCR was also demonstrated in a white-rot fungus, *Dichomitus squalens* [26], a species closely related to *Ganoderma*. Analysis of the transcriptome and proteome data showed that presence of glucose repressed the expression of ~ 7% genes, primarily CAZymes and carbon catabolic genes, in *D. squalens* [26]. In the current DEGs dataset, 28 (80%) of the *in planta* expressed glycosyl hydrolases, that target cellulose and hemicellulose components of plant cell wall, were down-regulated when *G. boninense* was grown on artificial media, a relatively simple carbon source as compared to palm roots. Understanding the CCR mechanism in white-rot fungi would facilitate the development of modified strains with improved CAZyme transcription profile needed for biofuel production.

White-rot fungi preferentially remove lignin [47] leaving cellulose as the primary constituent of cells in delignified plant cell walls [48]. Removal of lignin provides easy access to cellulose and hemicellulose that are processed into oligo- and monosaccharides [49]. Thus, breakdown of plant cell wall requires concerted effort from different enzyme families specializing in targeting specific components [49]. A number of *G. boninense* carbohydrate active enzymes (CAZymes) were up-regulated during colonization of palm lignocellulosic substrate. Certain CAZymes, like, GH3, GH28, LPMO (AA9, formerly GH61), PODs (class II peroxidases) and MCOs (multi copper oxidases) that have expanded in white-rot fungi [50] were up-regulated *in planta* in this interaction. The CAZymes up-regulated early in the infection process in another white-rot fungus, *Heterobasidion irregulare*, such as, pectin degrading enzymes (polysaccharide lyases and GH28) cell wall targeting enzymes (GH1, GH61) and enzymes with CBM1 modules [51], were also found to be up-regulated in *G. boninense*. In fungi, especially phytopathogens, the CAZyme profiles may be tailored to the cell wall composition of their respective monocot or dicot hosts [52]. The enzyme profile of *G. boninense* during growth on different carbon sources would help determine if it has the capacity to colonize substrates beyond those typically associated with monocots.

Auxiliary enzymes are essential for facilitating the cell wall degradation process even though these cannot breakdown lignocellulosic compounds. One large gene family with auxiliary function in lignin modification is the Cytochrome P450s that detoxify the aromatic byproducts resulting from lignin breakdown [53]. Another auxiliary biochemical process facilitating lignin degradation is the halogenation and dehalogenation of aromatic rings. Chloroperoxidases were shown to chlorinate (halogenate) and cleave lignin components [54]. The halogenated aromatic rings may then become substrates for haloacid dehalogenase (HAD) enzymes that split carbon-halogen bonds and play an auxiliary role in lignin depolymerization. The *in planta* concerted expression of CAZymes, auxiliary enzymes like Cytochrome P450s and HADs, other genes involved in oxalic acid degradation and transporter proteins involved in shuttling toxic by-products and maintaining cellular homeostasis, suggests that *G. boninense* was actively involved in cellular decay when the plant tissue was sampled one-month post inoculation.

Oxalic acid plays a role in pathogenesis in necrotrophic fungi [55] as well as promoting degradation of wood lignin in white-rot fungi [56]. But as oxalic acid is toxic, specific genes like oxalate decarboxylases (ODCs) belonging to the bicupin family and oxalate oxidases are needed for its degradation [56]. Another class of genes, formate dehydrogenases (FDHs), that breakdown the formate generated by the ODC genes [56] were also up-regulated. The up-regulation of oxalate breakdown genes coupled with lack of oxalate biosynthesis gene expression in *G. boninense* suggests that it could also be a response to counteract oxalate production by the host.

This study provides a glimpse into the transcriptional changes that occur in *G. boninense* as it interacts with oil palm roots. Another *Ganoderma* species described on palms that is relevant in North American landscapes, *G. zonatum*, groups with its sister species, *G. boninense*, in clade C [6]. It remains to be seen whether convergent or divergent evolution played a role in the origin of host preference for palms in these two lineages of *Ganoderma*.

## Conclusions

This analysis identified *Ganoderma boninense* genes that facilitate the colonization of roots of its oil palm host, *Elaeis guineensis*. The expression of a number of CAZymes involved in plant cell wall degradation and other auxiliary enzymes, such cytochrome P450s and HAD proteins, that function to breakdown byproducts of degradation were up-regulated during the interaction. Several *G. boninense* pathogenicity-related genes and those responsible for oxidative phosphorylation and mitochondrial biogenesis were identified as down-regulated *in planta*. In conclusion, this examination of *G. boninense*-oil palm interaction offers a prelude to understanding how white-rot fungi in the genus *Ganoderma* colonize palms.

## Methods

### Data retrieval

Genome sequence for *Ganoderma boninense* isolate G3, collected from North Sumatra province in Indonesia, was downloaded from NCBI (BioProject PRJNA421251; [13]). The interaction of *G. boninense* isolate PER71 with oil palm, *Elaeis guineensis*, was sampled at a single time point, one-month post inoculation [14]. Briefly, the experiment was comprised of two growth conditions: 1) in vitro - *G. boninense* culture grown on artificial media with three replicates (SRA samples, SRR8432491-SRR8432493), and 2) *in planta* - *E. guineensis* roots inoculated with *G. boninense* colonized rubberwood blocks in three replicates (SRA samples, SRR8432494-SRR8432496). RNA was isolated from fungal cultures and roots of five-month infected oil palm seedlings and sequenced on the Illumina HiSeq 1000 platform. The data for these six samples was downloaded from NCBI (BioProject PRJNA514399; [14]).

### RNAseq analysis

**Fig. 5:**
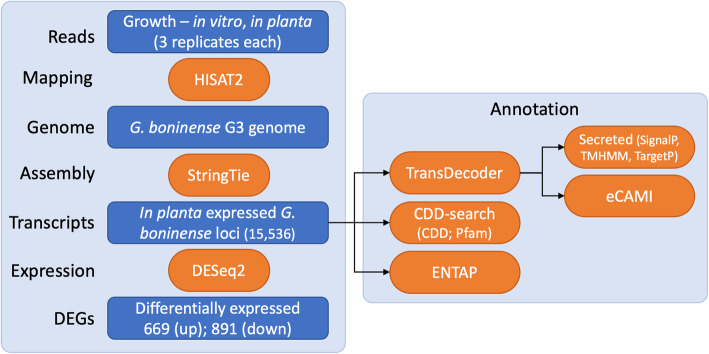
Identification and annotation of transcripts and differentially expressed genes (DEGs). A schematic for the pipeline used to generate transcript loci and identify differentially expressed genes (DEGs). The assembled transcripts were annotated both at the nucleotide and protein level

A data analysis pipeline was built to identify and characterize the RNAseq dataset (Fig. 5). The reads for the six samples downloaded from NCBI were mapped to the unmasked *G. boninense* genome using the splice-aware aligner HISAT2 ver.2.1.0 [57]. The resulting mapping files (SAM format) for each sample were converted to compressed BAM format and sorted using SAMtools ver.1.9 [58] before doing transcript assembly with StringTie ver.2.0.6 [59] (Additional file 2: Dataset S1) and estimating raw counts for transcript abundance. The raw counts matrix was formatted using ‘prepDE.py’, a python script provided with StringTie, and used as input for differential gene expression analysis with R v.3.6.3 package DESeq2 [60]. For each transcript locus, normalized read counts along with fold change and false discovery rate (FDR)-adjusted *p*-values (p_adj_) were obtained from DESeq2. Three criteria were used to define a set of differentially expressed genes (DEGs), i) absolute value [log_2_FC (fold change) > 2], ii) *p*-value adjusted for multiple testing, p_adj_ < 0.05, and iii) non-zero normalized count values across all treatments. Heatmaps for DEGs were generated using R v.3.6.3 package pheatmap v.1.0.12 [61].

### Annotation

The StringTie assembled transcript sequences (Additional file 3: Dataset S2) were annotated using the Reverse Position-Specific BLAST (RPS-BLAST) tool [15] from NCBI. RPS-BLAST utilizes a database of pre-computed Position-Specific Score Matrix (PSSM) to identify protein domains in either nucleotide or protein query sequences. A standalone version of RPS-BLAST, also known as CD-Search (Conserved Domain Search), along with ‘rpsbproc’ a command line utility to process RPS-BLAST results, were downloaded from NCBI FTP ftp://ftp.ncbi.nlm.nih.gov/pub/mmdb/cdd/. Two pre-formatted protein domain databases, NCBI Conserved Domain Database (CDD [62];) and Pfam [63], containing PSSMs were accessed from the above FTP service and used to search the StringTie derived transcript dataset. Protein domain name and descriptions were transferred to the transcripts based on the CD-Search results. Additionally, ENTAP (Eukaryotic Non-Model Transcriptome Annotation Pipeline [64];) was used to annotate the complete set of assembled transcripts. The ENTAP pipeline utilized DIAMOND [65] for protein alignment and three databases, NCBI nr (v4), Uniprot (release-2020_01) and fungal sequences from NCBI RefSeq (RefSeq-release200.txt) were used to annotate proteins. The CD-Search and ENTAP output for the DEGs was compared manually. A discrepancy in the output from the two pipelines, if any, was resolved by comparing to NCBI BLAST search results. The results for a small percentage (~ 2%) of DEGs were also manually curated using NCBI BLAST.

The assembled transcripts were translated to proteins using TransDecoder-ver.5.5.0 [66]. Two flags, a minimum protein length of 50 amino acids (−m 50) and ‘--single_best_only’ were used. The resulting protein dataset was parsed to retain proteins that started with a Methionine at the N-terminus (Additional file 4: Dataset S3). A simple custom pipeline was used to predict secreted proteins from the parsed protein dataset. Briefly, proteins with a signal peptide (signalP-5.0b; [67]) that lacked a transmembrane helix (tmhmm-2.0c; [68]) and targeted to secretory pathway (targetP-2.0; [69]) were considered as secreted proteins. Carbohydrate active enzymes (CAZymes) were predicted by using a k-mer based tool called eCAMI (simultaneous enzyme Classification And Motif Identification; [70]) that compared the parsed protein dataset against the CAZy database [71].

## Supplementary Information

**Additional file 1:** **Table S1.** The expression, annotation and coordinates of *G. boninense* transcripts. Normalized expression values, log2 fold-change and p-adj values for 15,536 transcript loci are listed in this table. Protein domain names and descriptions obtained from two databases, CDD and Pfam, and results from three pipelines, ENTAP, secreted protein and eCAMI (CAZymes) are presented for the 1560 differentially expressed genes. Value ‘YES’ in ENTAP column means that the annotation was manually curated (last column). The values with an asterisk in the CAZyme column were results obtained from other annotation sources. CDD and Pfam domain names, and results from CAZyme and secretion signal detection pipeline are included for the remaining transcript loci.

**Additional file 2:** **Dataset S1.** StringTie transcript gtf annotation.

**Additional file 3:** **Dataset S2.** StringTie assembled transcripts.

**Additional file 4:** **Dataset S3.** TransDecoder derived proteins.

## Data Availability

The data generated in this study are included in this manuscript and additional files. The reference genome assembly used for data analysis was obtained from National Center for Biotechnology Information (NCBI) BioProject PRJNA421251 and can be accessed from this link, https://www.ncbi.nlm.nih.gov/nuccore/PJEW00000000. The raw transcriptome data used in the analysis can be obtained from NCBI BioProject PRJNA514399, and can be downloaded from the following links: https://sra-downloadb.be-md.ncbi.nlm.nih.gov/sos1/sra-pub-run-2/SRR8432491/SRR8432491.1 https://sra-downloadb.be-md.ncbi.nlm.nih.gov/sos1/sra-pub-run-2/SRR8432492/SRR8432492.1 https://sra-downloadb.be-md.ncbi.nlm.nih.gov/sos1/sra-pub-run-2/SRR8432493/SRR8432493.1 https://sra-downloadb.be-md.ncbi.nlm.nih.gov/sos1/sra-pub-run-2/SRR8432494/SRR8432494.1 https://sra-downloadb.be-md.ncbi.nlm.nih.gov/sos1/sra-pub-run-2/SRR8432495/SRR8432495.1 https://sra-downloadb.be-md.ncbi.nlm.nih.gov/sos1/sra-pub-run-2/SRR8432496/SRR8432496.1

## Abbreviations

Nr: Non-redundant database
NCBI: National Center for Biotechnology Information
CDD: Conserved domain database
Pfam: Protein family
GDP: Gross domestic product
CCR: Carbon catabolite repression
DEGs: Differentially expressed genes
CAZYmes: Carbohydrate-Active enZYmes
GH: Glycoside hydrolase
GT: Glycosyl transferase
CBM: Carbohydrate binding module
PL: Polysaccharide lyase
CE: Carbohydrate esterase
ABC: ATP binding cassette
HAD: Haloacid dehalogenase
ODC: Oxalate decarboxylase
FDH: Formate dehydrogenase
LPMO: Lytic polysaccharide monooxygenase
POD: Peroxidase
MCO: Multi copper oxidase
CHS: Chitin synthase
Hce2: Homologs of *C. fulvum* Ecp2
CFEM: Common in several fungal extracellular membrane protein
CAP: Cysteine-rich secretory proteins, antigen 5, and pathogenesis-related 1 proteins
AOX: Alternative oxidase
MFS: Major facilitator superfamily
CoA: Coenzyme A
ATP: Adenosine triphosphate
MAM33: Mitochondrial acidic matrix protein
MAMP: Microbe-associated molecular pattern
PX: Phox domain
